# Supplementary material for: MetaRibo-Seq measures translation in microbiomes
Source: Nat Commun. 2020 Jun 29;11:3268. doi: 10.1038/s41467-020-17081-z (PMC7324362; doi:10.1038/s41467-020-17081-z)
Supplement: Supplementary file 10 — Supplementary Data 7 [file 41467_2020_17081_MOESM10_ESM.zip › File2/Confidence_High_Taxonomy/179757_out.krona.html]

Javascript must be enabled to view this page.

members
magnitude
magnitudeUnassigned
count
unassigned
taxon
rank

179757\_out


SRS053356\_contig\_number\_47574
7
1

2759
superkingdom
6

kingdom
4751
6

subkingdom
451864
6

5
5204
phylum

5
subphylum
5302

5
155619
class

5
452333
subclass

5
order
5338

5
862241
family

937605
genus
5

937743
species

SRS013687\_contig\_number\_10791SRS023914\_contig\_number\_contig-100\_316.156777SRS051031\_contig\_number\_36143SRS098571\_contig\_number\_62467SRS1041145\_contig\_number\_11054
5

4890
phylum
1

subphylum
147538
1

1
147550
class

1
subclass
222543

1
order
5125

1
family
110618

genus
140106
1


SRS076929\_contig\_number\_contig-100\_74.144073
1
78410
species
